# Growth Arrest on Inhibition of Nonsense-Mediated Decay Is Mediated by Noncoding RNA GAS5

**DOI:** 10.1155/2013/358015

**Published:** 2013-11-11

**Authors:** Mirna Mourtada-Maarabouni, Gwyn T. Williams

**Affiliations:** Institute for Science and Technology in Medicine and School of Life Sciences, Keele University, Huxley Building, Keele ST5 5BG, UK

## Abstract

Nonsense-mediated decay is a key RNA surveillance mechanism responsible for the rapid degradation of mRNAs containing premature termination codons and hence prevents the synthesis of truncated proteins. More recently, it has been shown that nonsense-mediated decay also has broader significance in controlling the expression of a significant proportion of the transcriptome. The importance of this mechanism to the mammalian cell is demonstrated by the observation that its inhibition causes growth arrest. The noncoding RNA growth arrest specific transcript 5 (GAS5) has recently been shown to play a key role in growth arrest induced by several mechanisms, including serum withdrawal and treatment with the mTOR inhibitor rapamycin. Here we show that inhibition of nonsense-mediated decay in several human lymphocyte cell lines causes growth arrest, and siRNA-mediated downregulation of GAS5 in these cells significantly alleviates the inhibitory effects observed. These observations hold true for inhibition of nonsense-mediated decay both through RNA interference and through pharmacological inhibition by aminoglycoside antibiotics gentamycin and G418. These studies have important implications for ototoxicity and nephrotoxicity caused by gentamycin and for the proposed use of NMD inhibition in treating genetic disease. This report further demonstrates the critical role played by GAS5 in the growth arrest of mammalian cells.

## 1. Introduction

GAS5 (growth arrest-specific transcript 5) was identified using a functional screen through its ability to suppress apoptosis in a mouse thymoma cell line [1]. This gene is encoded at 1q25, a chromosomal locus which has been associated both with leukaemia and lymphoma [2–4] and with systemic lupus erythematosus (SLE) [5–8].

GAS5 was initially isolated from a subtraction cDNA library as part of a strategy intended to identify genes enriched on growth arrest [9]. GAS5 encodes small nucleolar RNAs (snoRNAs) in its introns, and its exons contain a small open reading frame (ORF) which does not encode a functional protein [10]. The snoRNAs expressed from the intronic regions of GAS5 are involved in the biosynthesis and processing of ribosomal RNA, which has always been assumed to be an essentially housekeeping role. However, a number of lines of evidence have emerged recently which indicate the involvement of snoRNAs in regulating cell growth and proliferation [11]. Gene expression studies have shown a significant upregulation of GAS5 by oncogenic kinases associated with myeloproliferative disorders [12]. GAS5 is also involved in a chromosomal rearrangement with Notch 1 in radiation-induced thymic lymphoma [13]. Most importantly, GAS5 has been shown to play critical roles in normal growth arrest in both primary and transformed human cells [14, 15] and in the inhibition of human T-cell proliferation produced by mTOR antagonists such as rapamycin and its analogues [16].

GAS5 is transcribed as a 5′-terminal oligopyrimidine (5′TOP) RNA and thus belongs to a class of transcripts characterised by an oligopyrimidine tract sequence at its 5′ end. Other 5′TOP RNAs encode ribosomal proteins, as well as other proteins involved in protein synthesis (reviewed by Meyuhas and Dreazen [17]). 5′TOP transcripts share some distinctive characteristics in common, including the inhibition of their translation by the immunosuppressant rapamycin [18]. An additional characteristic of 5′TOP mRNAs is that they are subject to growth-dependent translational control, which explains the previously reported posttranscriptional accumulation of GAS5 mRNA in growth-arrested cells [19]. The complex processing of GAS5 transcripts results in the production of many different splice variants which are normally associated with ribosomes [19]. The open reading frame of human spliced GAS5 is small, and its termination codon is found in an early exon, suggesting that these transcripts are subject to nonsense-mediated decay (NMD) when translated [19, 20]. In growing cells, the active translation of all 5′TOP RNAs leads to rapid degradation of the GAS5 transcripts by NMD, whereas, in growth arrested cells, inhibition of translation would be expected to lead to the accumulation of GAS5 transcripts, since NMD only affects mRNAs which are being translated [19].

The NMD pathway is an essential process in cell growth and development. It acts as an RNA surveillance mechanism by promoting degradation of mRNAs containing premature stop codons [21] and also regulates the expression of a small but significant fraction of the cell's transcriptome [22]. Absence of NMD results in the accumulation of transcripts containing premature stop codons leading to the translation and stabilisation of truncated proteins, which have deleterious effects for the cell (reviewed by Brogna and Wen [23], and by Nicholson and Mühlemann [24]). The DNA and RNA helicase UPF1 (up-frameshift suppressor 1) plays a key role in NMD [25, 26], and consequently the depletion of UPF1 by RNAi inhibits NMD [27]. UPF1 has also been found to be essential for human cells to complete DNA replication and for genomic stability [28]. Since GAS5 is also important for the control of the survival and proliferation of lymphocytes [14] and its abundance within the cell is controlled by NMD, we set out to test the working hypothesis that the effects of UPF1 could be mediated in part through the regulation of GAS5 mRNA levels, using RNA interference to inhibit NMD by downregulating UPF1.

The aminoglycoside antibiotics G418 and gentamycin bind to ribosomes and interfere with chain elongation, so that, at high concentrations, they block protein synthesis, and, at lower concentrations, they inhibit NMD while protein synthesis continues [29, 30]. We therefore used these compounds at low concentrations as an independent strategy for inhibiting NMD.

## 2. Materials and Methods

### 2.1. Cell Culture

The B-lymphoblastoid cell line BJAB and the cloned human T-leukemic cell lines CEM-C7 (clone CKM1) and Jurkat (clone JKM1) were maintained in RPMI-1640 medium (Sigma) supplemented with 10% heat inactivated fetal calf serum (HyClone), 2 mM L-glutamine, at 37°C in a 5% CO_2_ humidified incubator.

### 2.2. Determination of Cell Viability

Cell viability was determined by the Live/Dead viability assay (Molecular probes; cat. no. 03224). 200 *µ*L of cells (2 × 10^5^ cells/mL) was incubated in 96 well plates for 48 hours. An aliquot of the control or treated cells was added to 100 mL of the combined Live/Dead assay reagents (as instructed by the manufacturer). Cells were then incubated for 40 minutes at room temperature. Live cells stained with the green fluorescent dye and dead cells stained with the red fluorescent dye were visualised and counted using a Nikon Eclipse E400 fluorescence microscope.

### 2.3. DNA Assay (5-Bromo 2′-Deoxyuridine (BrdU) Incorporation)

The effects of UPF1 and GAS5 down-regulation on the proliferation of CEM-C7, Jurkat, and BJAB cells were assessed by bromodeoxyuridine (BrdU) incorporation during DNA synthesis using a colorimetric ELISA Kit (Roche Diagnostics, Germany; cat. no. 11647229001), following the manufacturer's instructions. In brief, 200 *μ*L cells (2 × 10^5^ cells/mL) was cultured in flat-bottom 96-well plates for 48 h. Subsequent to labelling with 10 *μ*M of BrdU (for the final 18 h of the incubation period), DNA was denatured and cells were incubated with anti-BrdU monoclonal antibody, prior to the addition of substrate. The absorbance of the samples was measured using a microplate reader (Wallac 1420 Victor Plate Reader) at 450 nm with the absorbance at 690 nm as reference.

### 2.4. Clonogenic Assay

Long-term survival of transfected cells treated with G418 (Invitrogen) or gentamycin (Sigma) was assessed by the ability of the cells to form colonies in soft agar. An equal proportion of culture from each experimental condition was diluted in 5 mL Iscove's medium (Sigma) containing 20% heat inactivated fetal calf serum, 10% cell-conditioned medium, and 0.3% noble agar (Difco) and plated in 60 mm dishes, overlaid with 2.5 mL Iscove's complete medium containing 10% cell conditioned medium. Colonies were counted following 2-3 weeks incubation at 37°C in 5% CO_2_ and 95% air.

### 2.5. RNA Interference

Transfection of UPF1, GAS5, and control siRNAs was as previously described [14]. Three different GAS5 siRNAs (small interfering RNAs) were designed by Ambion (siRNAs id 290458 (GAS5siRNA2); 290460 (GAS5siRNA1), 290459 (GAS5siRNA3); reference sequence AF141346). Three different UPF1 siRNAs (siRNAs id 12379 (UPF1 siRNA1); 142478 (UPF1 siRNA2); 12197 (siRNA3)) were also designed by Ambion. Negative control siRNA ((−)siRNA cat. no. 4605) was also purchased from Ambion. All siRNAs were purchased, already HPLC purified, annealed, and ready to use. To analyse the siRNA transfection efficiency, siRNA duplexes were labelled with Cy3 using the *Silencer* siRNA labelling kit (Ambion; cat. no. 1632), following the manufacturer's instructions, and transfection efficiencies (fluorescently labelled cells after 48 h) were 70%–80%. On the day before transfection, cells were split and cultured in RPMI supplemented with 10% FCS. On the day of transfection, 10^6^ cells (CEM-C7, Jurkat or BJAB) were centrifuged and washed once in Opti-MEM 1 (Invitrogen; no. 51985-026) before resuspension in 400 *μ*L Optimem. Cells were then incubated with 20 nM or 100 nM siRNA duplex for 10 minutes at room temperature in a 0.4 cm electroporation gap cuvette. Cells were electroporated for 25 milliseconds at 248 V (CEM-C7) or 293 V (Jurkat and BJAB) 1050 *μ*F using a Biorad Gene Pulser. Following electroporation, cells were incubated at room temperature for 20 min prior to transfer to 6 well plates containing Iscove's medium (Sigma) supplemented with 2 mM glutamine and 20% heat-inactivated FCS. The analysis of specific silencing of GAS5 expression was carried out after 48 hours, using real-time RT-PCR.

### 2.6. Real Time RT-PCR

Real-time RT-PCR was performed using 2 *μ*L of the cDNA prepared as described for RT-PCR above (equivalent to 500 ng of the total RNA) and TaqMan MGB probes and primers specific to human UPF1 (Applied Biosystems; assay id Hs00161289_m1) and human GAS5 (Exon 12: designed by Applied Biosystems; Forward primer CTTCTGGGCTCAAGTGATCCT; Reverse primer TTGTGCCATGAGACTCCATCAG; reporter CCTCCCAGTGGTCTTT) with eukaryotic 18S rRNA as an endogenous control (Applied Biosystems; assay id Hs99999901_s1), according to the manufacturer's instructions. Quantitation of GAS5 and UPF1 in cells transfected with GAS5 and UPF1 siRNAs constructs relative to (−)siRNA-transfected cells was determined using the comparative *C*_*T*_ method, using untransfected cells as calibrators. The ABI Prism 7000 sequence detection system was used to measure real-time fluorescence, and data analysis was performed using ABI Prism 7000 SDS software.

### 2.7. Statistical Analysis

Data are presented as the mean ± standard error of the mean (s.e.m.). Statistical significance was determined by analysis of variance using Origin 6.1. A *P* value of <0.01 was considered statistically significant.

## 3. Results

### 3.1. Downregulation of UPF1 Reduces Cell Viability and Inhibits Cell Proliferation

**Figure 1:**
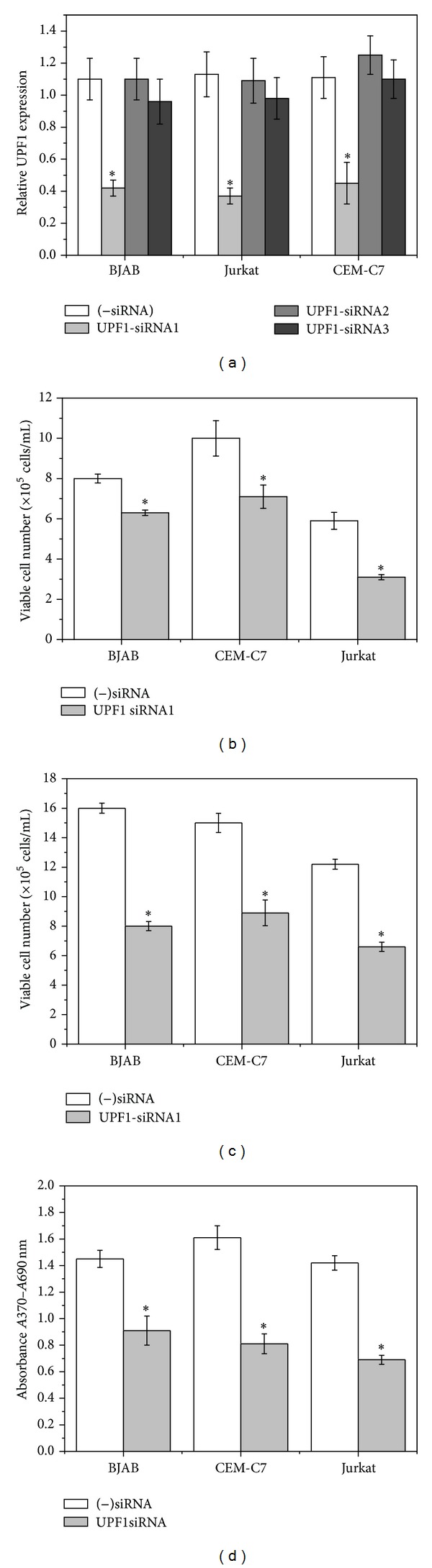
UPF1-specific siRNA increases cell death and inhibits cell proliferation in the human T-cell lines Jurkat and CEM-C7 and in the B-lymphoblastoid cell line BJAB. (a) Cells were transfected with 3 different specific UPF1 siRNAs or negative control siRNA ((−)siRNA) and cultured at 37°C. After 72 h the expression of endogenous UPF1 in the siRNA transfected cells was determined by real-time RT-PCR (mean ± s.e.m. from three separate experiments) and expressed relative to untransfected controls. ((b)–(d)) Cells were transfected with UPF1-siRNA1 or negative control siRNA ((−)siRNA) and cultured at 37°C. Viable cell numbers were determined after 48 h (b) and 72 h (c) by the LIVE/DEAD assay (Section 2.2). Results are represented as mean ± s.e.m. from three independent experiments. (d) Cell proliferation was measured after 48 h using the BrdU colorimetric ELISA assay. Results are represented as mean ± s.e.m. from three independent experiments. *P < 0.01 compared with (−)siRNA.

Initial experiments were carried out to study the physiological importance of UPF1 in the human B-cell line BJAB and the T-cell lines Jurkat and CEM-C7, which all proliferate continuously in culture without stimulation. We therefore used RNA interference (RNAi) to investigate the effects of downregulation of endogenous *UPF1 *expression in these cells. Transfecting the cells with UPF1 siRNA1 caused downregulation of UPF1 by 60%–65%, as shown in Figure 1(a) (the other 2 siRNAs tested were not effective). Downregulation of UPF1 caused a substantial reduction in viability of cell populations in the three cell lines at 48 h and 72 h (Figures 1(b) and 1(c)). Cell proliferation of BJAB, CEM-C7, and Jurkat cells was also inhibited as a result of UPF1 down-regulation (Figure 1(d)).

### 3.2. The Inhibitory Effect of UPF1 on Cell Viability and Growth Is Mediated through an Increase in Endogenous GAS5 mRNA Levels

**Figure 2:**
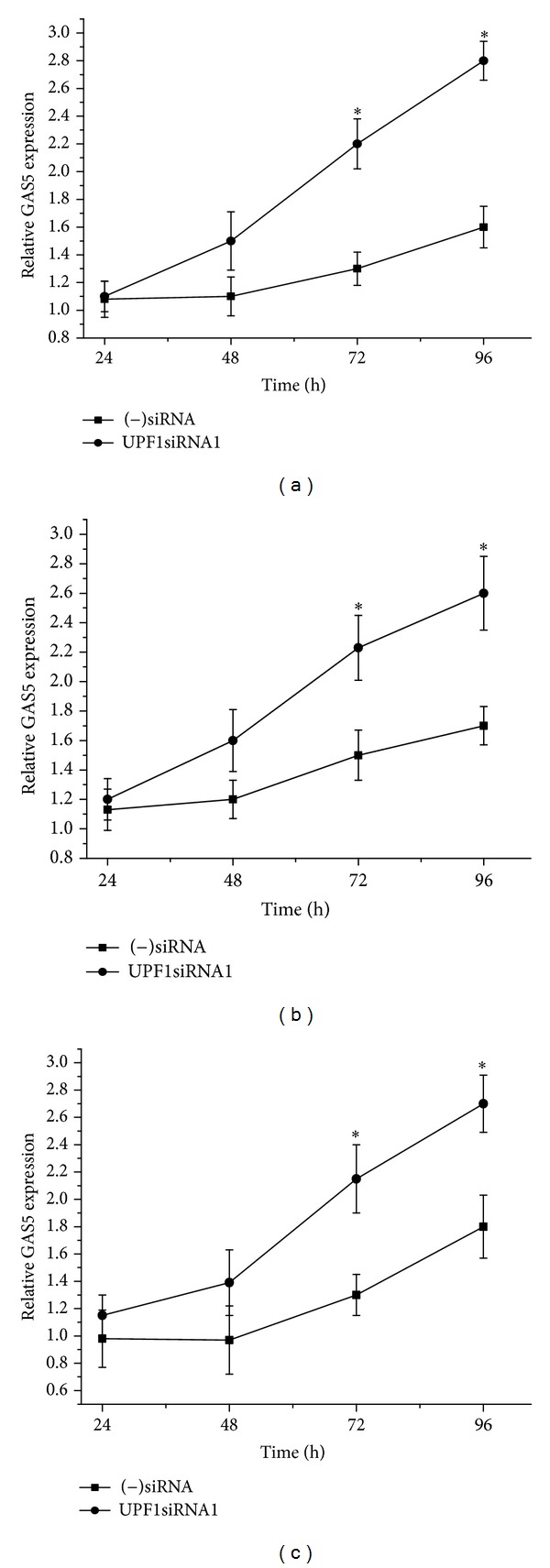
Down-regulation of UPF1 increases GAS5 mRNA levels. The human T-cell lines Jurkat and CEM-C7 and in the B-lymphoblastoid cell line BJAB were transfected with specific UPF1siRNA1 or negative control siRNA ((−)siRNA) and cultured at 37°C. 48 h, 72 h, and 96 h after transfection, and the expression of endogenous GAS5 mRNA in the siRNA transfected BJAB (a), Jurkat (b), and CEM-C7 (c) was determined by real-time RT-PCR using the comparative *C*_*T*_ method, normalised with 18S RNA as an internal control. The results are represented as the mean ± s.e.m. from three separate experiments. *P < 0.01 compared with (–)siRNA.

Previous work has shown that depletion of UPF1 in Hela cells was accompanied by an increase in the levels of GAS5 transcripts [31]. We therefore investigated the level of GAS5 in UPF1siRNA-transfected lymphoid cells. Down-regulation of UPF1 in the three cell lines, BJAB (Figure 2(a)), Jurkat (Figure 2(b)), and CEM-C7 (Figure 2(c)), led to significant accumulation of endogenous GAS5 mRNA 48 h and 72 h after transfection, as confirmed by qRT-PCR (Figure 2).

**Figure 3:**
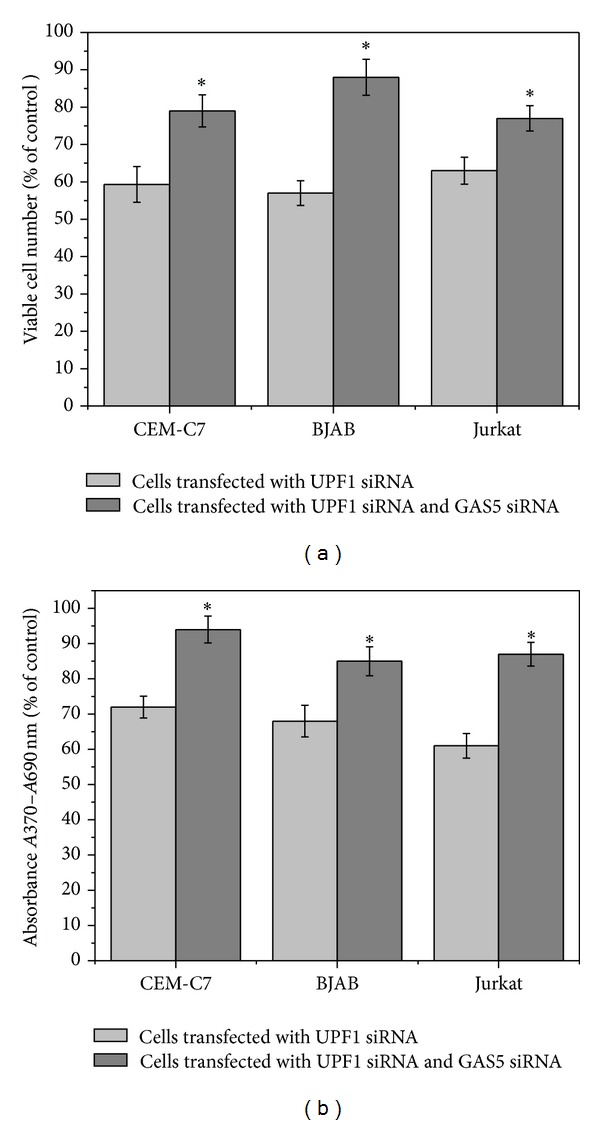
Down-regulation of GAS5 alleviates the inhibitory effects of UPF1 siRNA on cell viability and proliferation. CEM-C7, Jurkat, and BJAB cells were transfected with either control (−)siRNA or UPF1-siRNA1. 24 h after transfection, control cells and cells transfected with UPF1 siRNA were transfected with (−)siRNA or GAS5 siRNAs. Viable cell number (a) and cell proliferation (b) were determined after 72 h. Results are calculated as percentage viable cell number and absorbance relative to controls transfected with (−)siRNA. Data represent means ± s.e.m. from three independent experiments. *P < 0.01 compared to UPF1 siRNA, (−)siRNA1-transfected cells.

Previous results have shown that overexpression of GAS5 causes both an increase in cell death and a reduction in the rate of progression through the cell cycle both in T-cell lines and in untransformed human peripheral blood T-cells [14], producing effects which are somewhat similar to the effects observed with UPF1 downregulation (Figures 1(b), 1(c), and 1(d); Azzalin and Lingner, 2006 [28]). Since UPF1 down-regulation results in the accumulation of GAS5 mRNA (Figure 2), our working hypothesis was that the inhibitory effects of UPF1 on cell viability and growth could be mediated, to some extent, through the increase in endogenous GAS5 mRNA levels. Using GAS5-specific siRNAs [14], we studied the effects of GAS5 downregulation on the viability of the cells transfected with UPF1 siRNA. CEM-C7, Jurkat, and BJAB cells were transfected with UPF1-specific siRNA and negative control siRNA ((−)siRNA). 24 h after transfection, control cells (transfected with negative control siRNA) and cells transfected with UPF1 siRNA were transfected with (−)siRNA or GAS5-specific siRNAs (downregulation of 60%–65% for siRNA targeting UPF1 and 65%–73% for all three siRNAs targeting *GAS5* was confirmed by quantitative RT-PCR; data not shown). As similar results were obtained from all three GAS5 siRNAs, data obtained using only one GAS5 siRNA are shown. As shown previously [14], knockdown of GAS5 in BJAB, Jurkat, and CEM-C7 cells caused some increase both in viable cell number (Figure 3(a)) and in cell proliferation (Figure 3(b)). Down-regulation of UPF1 caused a decrease in the number of viable cells in BJAB, Jurkat, and CEM-C7 cells (Figure 3(a)) and a clear reduction in cell proliferation in all cell types (Figure 3(b)), confirming the results obtained earlier. However, GAS5-specific siRNA significantly alleviated the effects of UPF1 down-regulation in all three cell lines (Figures 3(a) and 3(b)), indicating that the increase in cell death and inhibition of cell proliferation mediated by UPF1 down-regulation is mediated, at least in part, by the accumulation of GAS5 mRNA levels. Since the transfection efficiency was 70% to 80%, the true protective effect of GAS5 down-regulation is likely to be even greater than demonstrated here.

### 3.3. Downregulation of Endogenous GAS5 Reverses the Inhibitory Effects of Aminoglycoside Antibiotics Gentamycin and G418

**Figure 4:**
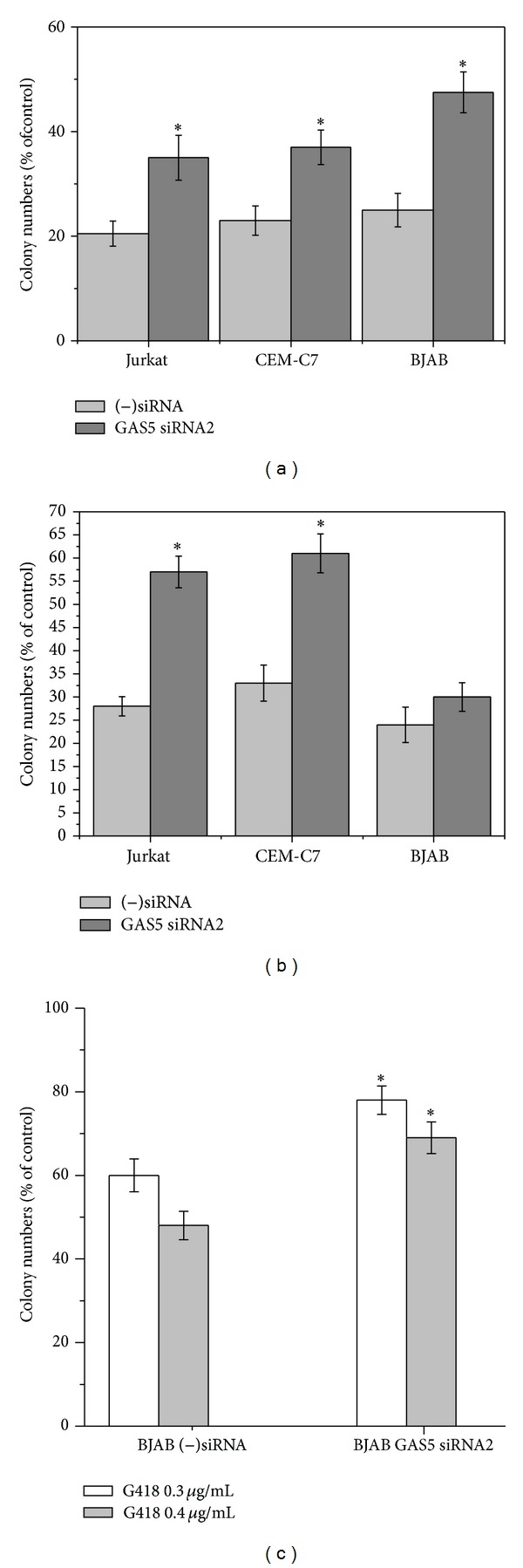
Down-regulation of GAS5 alleviates the inhibitory effects of aminoglycoside antibiotics on the colony forming ability of the human T-cell lines Jurkat and CEM-C7 and the B-lymphoblastoid cell line BJAB. CEM-C7, Jurkat, and BJAB cells were transfected with either control (−)siRNA or GAS5 siRNA2. 48 h after transfection, transfected cells were treated with gentamycin (100 *μ*g/mL) (a), G418 (0.5 *μ*g/mL) (b), or G418 (0.3–0.5 *μ*g/mL) on BJAB cells (c) for 72 h. Colony forming ability was then determined by plating in soft agar. Results are calculated as percentage colony numbers relative to controls incubated in the absence of aminoglycoside antibiotics. Means ± s.e.m. from four independent experiments are shown. *P < 0.01 compared to (−)siRNA.

It is well established that aminoglycoside antibiotics such as gentamycin and G418 can induce translational read-through of nonsense codons and thus suppress NMD [29, 30]. We therefore investigated whether down-regulation of GAS5 has any effect on the growth inhibitory effects of gentamycin and G418. CEM-C7, Jurkat, and BJAB cells were transfected with GAS5 siRNA2 and negative control siRNA. 48 h after transfection, control cells (transfected with negative control siRNA ((−)siRNA) and cells transfected with GAS5 siRNA2 (down-regulation of 65%–73%, as confirmed by quantitative RT-PCR; data not shown) were treated with G418 or gentamycin. As Figure 4(a) shows, GAS5-siRNA significantly protected colony-forming ability after treatment with gentamycin (1.7- to 2-fold) in Jurkat (1.7-fold), CEM-C7 (1.6-fold), and BJAB (1.9-fold) cells. Downregulation of GAS5 significantly alleviated the inhibitory effects of G418 (0.5 *μ*g/mL) on colony forming ability in CEM-C7 (2-fold) and Jurkat cells (1.9-fold) but the alleviation is not statistically significant at this concentration of G418 for BJAB cells (Figure 4(b)). Further experiments with lower concentrations showed that transfecting BJAB cells with GAS5 siRNA2 did significantly protect against the loss of colony forming ability induced by lower concentrations of G418 (0.3 and 0.4 *μ*g/mL; Figure 4(c); *P*, 0.01).

## 4. Discussion

NMD is one of the best characterized RNA surveillance mechanisms; it protects the cell from the potentially detrimental effects of truncated proteins by detecting mRNA transcripts containing premature termination codons in their ORF and committing them to rapid decay (reviewed by Brogna and Wen [23] and Nicholson and Mühlemann [24]). The well-conserved DNA and RNA helicase UPF1 is a crucial component of the core NMD machinery, and expression of a dominant-negative human UPF1 mutant, which contains a point mutation (R844C) in its RNA helicase domain, has been shown to impair NMD activation [32]. In addition, loss of UPF1 in a number of human cell lines has been reported to lead to impairment of NMD and to inhibition of cell proliferation [28]. In the present study, down-regulation of UPF1 caused a marked decrease in viability and proliferation of cell populations in the three cell lines examined (Figure 1). All these observations strongly suggest that UPF1 plays an important role in regulating the survival and proliferation of human lymphoid cell lines. These data are consistent with the observations that genetic disruption of the murine ortholog of UPF1 (RENT1) is embryonically lethal [33] (Medghalchi et al., 2001) and that mutation in UPF1 is lethal in Drosophila [26].

In Hela cells, depletion of hUPF1 caused an increase in the levels of GAS5 transcripts [31]. In addition, knockdown of SMG1, 8 and 9 (forming part of the SMG1 complex, the phosphatidylinositol 3-kinase that regulates the phosphorylation state and function of UPF1), also resulted in an accumulation of GAS5 mRNA [34]. The present work also shows that down-regulation of UPF1 caused a significant accumulation of endogenous GAS5 mRNA in all the cell lines studied (Figure 2). Our results also indicate that, in both T-cell lines and B-cell lines, the inhibitory effects of UPF1 on cell viability and growth are, at least in part, mediated through the increase in endogenous GAS5 mRNA levels, since down-regulation of GAS5 significantly alleviated the effects of UPF1 down-regulation (Figure 3).

Of all the factors which play essential roles in NMD [35], UPF1 is functionally the most important and has also been reported to have additional functions independent of NMD. UPF1 is involved in the degradation of replication-dependent histone mRNA upon replication inhibition and at the end of S phase [36] and is essential for S phase progression [28]. The present study demonstrates the importance of UPF1 in regulating the growth and survival of human lymphocytes and provides evidence that a substantial part of the antiproliferative effects of inhibition of NMD is mediated through GAS5. Given the established importance of GAS5 in the control of lymphoid cell proliferation and survival and the accumulation of GAS5 mRNA on UPF1 down-regulation, the alleviation of the effects of UPF1 knockdown by downregulating GAS5 indicates that the modulation of GAS5 transcript levels connects NMD to the control of cell division and survival. On the other hand, the incomplete rescue by GAS5 siRNAs (Figure 3) indicates that additional genes must also be involved.

At concentrations below those which block protein synthesis, aminoglycoside antibiotics gentamicin and G418 inhibit NMD by allowing read-through of stop codons [29, 30]. This independent strategy of pharmacological inhibition of NMD also results in a reduction in colony-forming ability, and these effects were also substantially alleviated by down-regulation of GAS5 (Figure 4). This provides important independent evidence supporting the proposed role of GAS5 in the cytotoxic and cytostatic effects of NMD inhibition. As GAS5 is only one of many genes which are affected by inhibition of NMD, such inhibition is likely to be the most important effect of these aminoglycoside antibiotics at the lowest lethal levels.

The effects of aminoglycoside antibiotics on NMD have two important clinical implications. Firstly, ototoxicity and nephrotoxicity are important side effects of clinical treatment with gentamicin [37, 38]. The present report suggests that, at the relatively low concentrations likely to be encountered in vivo, the cytotoxic effects observed on treatment with these antibiotics may be mediated by inhibition of NMD and the consequent increase in GAS5 levels. Secondly, inhibition of NMD has been proposed as a clinical treatment for genetic abnormalities, such as a proportion of cystic fibrosis cases, that are caused by nonsense mutations producing premature termination codons within the coding sequence (reviewed by Bhuvanagiri et al. [39]). The present report suggests that pharmacological inhibition of NMD as a clinical treatment will need to employ specific targeting strategies [39] rather than blanket inhibition of NMD in order to avoid the cytostatic and cytotoxic consequences of stimulation of GAS5 levels.

GAS5 RNA levels appear to be regulated primarily through changes in its rate of degradation rather than through changes in its rate of transcription [19, 40]. As for other 5' TOP RNAs, GAS5 mRNA stability is dependent on its translation, which is controlled, at least in part, by the mTOR pathway [18, 19], and on its degradation by the NMD pathway, as shown both previously [19, 20] and in the present study (Figure 2). Inhibition of mTOR and inhibition of NMD both inhibit cell proliferation, and in both cases, a substantial part of the inhibitory effect is mediated through GAS5 (Mourtada-Maarabouni et al. [16] and Figure 3), indicating that GAS5 frequently plays a critical role in growth arrest in mammalian cells.

Previous studies have highlighted the significance of changes in the level of GAS5 expression for the control of cell division and survival and demonstrated a crucial role for this noncoding RNA in the control of growth arrest, apoptosis, and the cell cycle [14–16]. In addition, GAS5 transcript levels were found to be significantly reduced in breast cancer samples relative to adjacent unaffected normal breast epithelial [15] and low tumour GAS5 expression has recently been associated with poor prognosis in patients with breast cancer and head and neck squamous cell carcinoma [41]. The remarkable effects of changes of GAS5 expression on the control of cell apoptosis and growth reported here and in other studies [14, 16] indicate the potential significance of this gene in the development and progression of cancer [11] and highlight the importance of further investigation into the role of GAS5 dysregulation in autoimmune disease and oncogenesis.
